# Searching for the Perfect Wave: The Effect of Radiofrequency Electromagnetic Fields on Cells

**DOI:** 10.3390/ijms15045366

**Published:** 2014-03-27

**Authors:** Lisa Gherardini, Gastone Ciuti, Selene Tognarelli, Caterina Cinti

**Affiliations:** 1 Institute of Clinical Physiology, Consiglio Nazionale delle Ricerche Siena, Strada Petriccio e Belriguardo, Siena 53100, Italy; E-Mail: lghera@gmail.com; 2 The BioRobotics Institute, Scuola Superiore Sant’Anna, Viale Rinaldo Piaggio 34, Pisa 56025, Italy; E-Mails: g.ciuti@sssup.it (G.C.); s.tognarelli@sssup.it (S.T.)

**Keywords:** radio frequencies, neurodegeneration, radiotoxicity, cancer

## Abstract

There is a growing concern in the population about the effects that environmental exposure to any source of “uncontrolled” radiation may have on public health. Anxiety arises from the controversial knowledge about the effect of electromagnetic field (EMF) exposure to cells and organisms but most of all concerning the possible causal relation to human diseases. Here we reviewed those *in vitro* and *in vivo* and epidemiological works that gave a new insight about the effect of radio frequency (RF) exposure, relating to intracellular molecular pathways that lead to biological and functional outcomes. It appears that a thorough application of standardized protocols is the key to reliable data acquisition and interpretation that could contribute a clearer picture for scientists and lay public. Moreover, specific tuning of experimental and clinical RF exposure might lead to beneficial health effects.

## 1. Introduction

For surfers all over the world, the perfect wave is a dream, a myth that will probably never materialize. But many of them know that to tame and control it is a matter of calculable strength, physics and power. Likewise, dealing with all the possible implications concerning electromagnetic field exposure is an exquisite matter of equilibrium between public health and social benefits. We set to review those recent reports that in our opinion added some new insight on the current knowledge about radiofrequency (RF) effects on a biological system. The mounting literature regarding the consequences of extremely low frequency-electromagnetic field (ELF-EMF) and RF exposure on cancer and the possible exploitation of RF in cancer treatment has been thoroughly reviewed elsewhere [1,2] and constitutes the basis for international and local regulatory authority guidelines for the environmental and commercial exposure to RF (National Institute of Health ISS) [3].

### 1.1. Who Is Afraid of “Electrosmog”?

Electromagnetic fields of all frequencies represent one of the most common environmental influences, and EMF exposure levels of the population will continue to increase with technological advances. Since the 1960s, anxiety and speculation has grown among the public about the consequence of casual exposure to or continuous interaction with different sources of EMF emission [4]. The neologism “electrosmog” is an indication of how this concern has spread among the population [5]. To answer such an alarmed call and prevent panic, the World Health Organization (WHO) promptly acted with dedicated topic sections for projects, initiatives and activities to provide reliable information to the lay public, which deals daily with any sort of uncensored information about possible detrimental effects of familiar devices and powered households believed to favor the onset of brain tumor or degenerative diseases.

The reality is that the level of RF radiation to which people is exposed is growing exponentially. This is suggested to correlate with an associated risk of glial tumor [6] Moreover, the specific use of several devices such as microwave ovens and mobile phones is today prevalent and some groups of study suggested that actual methods and parameters of evaluation of absorption underestimate the real impact of RF exposure. In these authors’ views, guideline limits of tolerability ought to be reevaluated, especially in the cases of youngsters and children [7].

To date, however, no clear answer has been given to rebut those skeptical voices and the ghost of the conflict of interest by multinational companies to hide the truth about the risks of EMF exposure.

### 1.2. Good, Bad, Ugly—or Simply Not Right?

To evaluate the potential consequences of EMF on public health, it is necessary to pay particular attention to the nature of those studies. Many of the recent studies are epidemiological or clinical examinations and adhere to different designs with respect to laboratory preclinical basic science; *in vitro vs. in vivo*. The methodology of data collection and observational reports is also important to account for possible variables that might bias the final outcome.

Our review intends to focus on the latest acquired information on the possible effects on biological systems of exposure to radiofrequency electromagnetic waves (RF-EMF) as the exposure to this range of radiations accompany the use of common devices such as mobile phones and Wi-Fi radiations. Until recently, experimental and epidemiological data for the RF range were very sparse and the results of numerous *in vitro* and *in vivo* studies were rather conflicting. In particular, there were discrepancies and diversities concerning the mechanistic explanation of the observed molecular events. This reflects the plethora of possible variables encountered in the evaluation of the effects of the EMFs on biological systems. To adopt a simplistic approach, one can consider that these effects depend on the frequency of the field and its intensities. However, several other parameters strictly depend upon factors such as the duration of exposure and the tissue penetration. Moreover, dealing with static or an oscillatory field induces a difference in the biological system response.

## 2. Technical Overview

The electromagnetic environment consists of natural radiation and manmade EMFs that are produced either intentionally or as by-products of the use of electrical devices and systems. Based on this, all populations are now exposed to varying degrees of EMFs, and the levels will continue to increase as technology advances.

EMFs are invisible areas of energy, often referred to as radiation, that are associated with the use of electrical power and various forms of natural (*i.e.*, electrical discharges in the earth’s atmosphere and radiation from sun and space) and manmade lighting [8]. These waves consist of oscillating electric and magnetic fields that influence each other and affect in different ways the biological systems (*i.e.*, cells, plants, animals and human beings). To better understand this reciprocal influence it is useful to start with an overall description of the physical properties of electromagnetic waves (EMWs).

EMWs are two waves in one: (i) an electrical wave creating an electrical field that moves along one plane; and (ii) a magnetic wave creating a magnetic field that moves perpendicularly to the electrical one.

EMWs can be characterized by their wavelength, frequency or energy, which are directly related to each other. The frequency of an EMW is defined as the number of times the electric or magnetic fields change their sign, at a given point, per time unit and it is measured in Hertz (Hz). The wavelength of an EMW is inversely proportional to the frequency of the wave, proportional to the speed of the wave in the medium (usually the medium is considered to be the vacuum, where the speed corresponds to the speed of light *c* = 3 × 10^8^ m/s). As an example, a typical microwave furnace emits a wave of 2.54 GHz, corresponding to a wavelength of 12 cm.

As a function of the frequency (or wavelength), the EMWs are usually classified into one of the two radioactive categories: (i) electromagnetic fields or non-ionizing radiations: Low-level radiation which is generally perceived as harmless to humans; and (ii) electromagnetic radiations or ionizing radiation, *i.e*., high-level radiation which has the potential for cellular and DNA damage. The ionizing radiations have a frequency high enough to produce the ionization of molecules and atoms through the breaking of the chemical bonds of the molecules. Examples of these radiations are the X-rays and radiations produced by radioactive sources. The non-ionizing radiations (NIR) correspond to those waves of the electromagnetic spectrum whose energy is too low to break the atomic bonds. Among these radiations are the ultraviolet and visible light, the infrared radiation, the radiofrequency and microwave emissions, the extremely low frequency fields and the static magnetic and electric fields. The NIR, even at very high intensity, cannot produce ionization in a biological system. Nevertheless, these radiations have been proven to produce other biological effects, such as heating, alteration of some chemical reactions or induction of electric currents on tissues and cells [9].

### 2.1. Radiofrequency Electromagnetic Fields (RF-EMF)

High frequency EMFs are part of the electromagnetic spectrum between the low frequency and the optical part of the spectrum. As this part of the spectrum is used for broadcasting and telecommunication, it is termed radio frequency (RF). The RF spectrum is defined in the frequency range between 9 kHz and 300 GHz. In this review, we will focus on the effect of frequencies close to 900 MHz. EMFs in this frequency range may have a continuous sinusoidal waveform, but more often they have a complex amplitude distribution over time. For example, for broadcast or telecommunication purposes they are modulated or pulsed. There are many sources of RF that we can encounter during everyday life. Among them, the most common (as a function of their emission frequency) are: (i) TV and PC monitors (3–30 kHz); (ii) amplitude modulated radio emitters (30 kHz–3 MHz); (iii) induction heaters for industry (0.3–3 MHz); (iv) thermo-shielding, diathermia chirurgic systems (3–30 MHz); (v) mobile telephones; (vi) microwave ovens, diathermia chirurgic systems (0.3–3 GHz); (vii) RADAR, satellite communications systems, microwave communications systems (3–30 GHz); and (viii) solar radiations (30–300 GHz).

The RF fields with frequency over 10 GHz are highly absorbed by the skin and a very small part of the energy can reach the internal organs of the human body. The dosimeter unity for these fields is the field intensity measured as power density (W/m^2^). Powers over 1000 W/m^2^ are necessary to produce harmful effects, such as skin burns or ocular cataracts with fields of frequency over 10 GHz. Such powers should only be found near powerful radars, but the presence of humans on those planes are prohibited by law. RF fields with frequencies in the range from 1 MHz to 10 GHz can penetrate exposed organic tissues and produce heating by absorption of the waves. The field’s penetration depth is a function of the field frequency, being bigger for the lower frequencies. The energy absorbed by the tissues and organs is measured in this case as a specific absorption coefficient in units of power per tissue mass (W/kg), which is the dosimeter unit in this case. To produce any harmful effect on human health, a specific absorption coefficient of 4 W/kg should be reached.

### 2.2. Safety Guidelines on Electromagnetics

Safety guidelines outline two different categories of restrictions for what concerns electromagnetic exposures, respectively named: (i) basic restrictions; and (ii) reference levels or maximum permissible exposure. The basic restrictions, that also include a proper safety factor, define the threshold values above which specific biological effects can be expected and thus occur. Effects can be tissue heating from RF energy absorption above 100 kHz or nerve stimulation, from contact currents or induced currents or fields in the body below 10 MHz. The RF energy absorption is limited by the specific absorption rate (SAR) averaged over the full body to prevent thermal stress, and over any 10 or 1 g tissue mass to prevent local thermal injuries. Below 10 MHz, the induced fields are additionally limited to prevent undesirable and hazardous excitation of nerve tissue. In other words, basic restrictions are defined in terms of the appropriate biologically effective quantities, and are set below the threshold for the appropriate critical effects. Since the direct measurement of the basic restrictions is technically difficult or often impractical in many daily situations, reference levels are derived from basic restrictions and they are derived and reported in terms of a directly measurable parameter of the external electromagnetic exposures; such a procedure makes the exposure guidelines more practical and versatile. Indeed, while the basic restrictions are closely related to the biological effect, the reference levels are directly related to the emission levels of the different electromagnetic sources and are thus easier to be evaluated [10,11].

Different scientific committees from national and international organizations develop the safety guidelines for electromagnetic field exposure. The most relevant of these organizations are the International Commission on Non-Ionizing Radiation Protection (ICNIRP) and the Standards Coordinating Committee 28 (SCC28) of the IEEE. These committees constantly monitor scientific literature and reports in order to tune exposure limits based on the effects that the scientific community has established. These limits, revised every few years, are issued in guidelines that are also revised every few years [12–14].

In the European Union, the 1998 ICNIRP Guidelines yield the basis for the national regulators to endorse appropriate legislation. In 2004, the European Union distributed a directive with the aim to guard workers from possible dangerous effects caused by electromagnetic fields [15]. The United States considers parts of both the guidelines of the National Council on Radiation Protection [16] and the 1992 issue of the guidelines of the IEEE C95.1 standard [14]. In Switzerland, national regulation uses ICNIRP Guidelines 1998, and was the first country to introduce precautionary limits below the ICNIRP guidelines for fixed installations in sensitive areas (e.g., schools, living areas, hospitals) which are 20 dB [17]. In the Commonwealth of Independent States (CIS), the exposure to electromagnetic fields is limited according to the Sanitary and Epidemiologic Rules and Regulations (the SanPiN [18,19]).

## 3. The Effect of RF Exposure on *in Vitro* Models: Finding the Pathways

It has long been speculated that mobile phone radiation (radiofrequency-modulated electromagnetic fields, RF-EMF) alters protein expression in human cell lines [20,21]. This does not mean that similar responses will take place in a human body exposed to this radiation. However, studying cell models is extremely helpful to evaluate genetic, proteomic, and phenotypic consequences to be expected by controlled acute or continued exposure to RF. *In vitro* studies concern cell kinetics and proliferation effects, effects on genes, signal transduction, alterations in membrane structure and function, and biophysical and biochemical mechanisms. In particular, a growing amount of data are emerging on the induction by RF on cell adaptive response (AR) that might protect those cells from concomitant hazards (*i.e.*, chemicals, ionizing radiations) causing lesions in the DNA. It is also important to consider the timing of exposure as well as the metabolic state of the cell during exposure, as proliferating active cells continuously repair DNA damage [22]. We will confine our review only to the latest findings, since accurate reviews on high throughput omic [23,24] and cell biology [25,26] analysis have been recently published [27].

### 3.1. Intracellular Pathways: The Usual Suspects

Early *in vitro* studies of RF-EMF were mostly designed to study the toxic effect of RF on cell. Some of them aimed at clarifying the intracellular pathways that would sustain induced DNA damage and cellular fate. For example, Bourthoumieu [28,29] demonstrated a genotoxic effect on amniotic cells exposed for 24 h (SAR 0.25 W/kg) to GSM-900 MHz. However, no direct cytogenic effect was detected after exposure. Recently, replicated dosing of a wider range of SAR (up to 4 W/kg) and similar experiments indicated that exposure did not affect the intracellular stress-related p53 pathway. p53 is an important checkpoint system which guards against genomic instability by inducing both arrest of the cell cycle and apoptosis. Previous data on short exposure of 900 MHz in lymphoblastic leukemia indicated activation of p53-related pathways. However, longer continuous exposure determined silencing of pro-apoptotic signals and activation of pro-surviving genes such as *Bcl-2* and *Ras* and *Akt1*. This dual effect can be reconciled as an early self-defense response triggered by DNA damage that confers to the survivor cells a further advantage to survive and proliferate [30]. In this case, the authors reported both genotoxic damage and changes in gene expression levels. To draw a complete picture of lymphoblastic leukemia response to RF exposure, further investigation was recently carried out by microarray analysis on cancer cells. Trivino Pardo and coworkers [31] confirmed that high frequency EMFs affect cellular systems by acting as genotoxic agents. Significant changes in expression levels of genes involved in DNA repair, cell cycle arrest, apoptosis, chromosomal organization, and angiogenesis were observed after both short- and long-term 900 MHz EMF exposure. DNA repairing machinery was found to be dually affected with activation of p53-related genes and down-regulation of down-strain effectors like BRCA2, XRCC3, and RFC1. Apoptotic signaling was affected by RF exposure, as APAF1 was found to be strongly over-expressed, while FASL and CASP8 were found to be down-regulated. Another component of “caspase” cascade, CASP10, was found to be up-regulated only at an early time. It appears that RF, acting as a genotoxic agent, quickly induces DNA damage by activating early on cell death effectors of apoptotic response. With respect to aggressiveness of the tumor cells, altered gene expression was detected for angiogenesis, differentiation and regulation of the cytoskeleton. In relation to genes involved in angiogenesis signaling, down-regulation of BAI1 and TNFSF15 was observed. Similarly, VEGFA, which acts in the permeability of endothelial cells, and FLT4 were inhibited at both MW-EMF exposure time, while EPO, IL8, STAT5B and VAV2, PGF, HPSE appear to be strongly activated. It appears therefore that in lymphoblastic leukemia cells, the 900 MHz MW-EMF may act as a double target negative regulator of genes: it affects the control of chromosomal organization and induces inhibition of angiogenesis that leads to tumor progression and metastatic transformation. It is worth mentioning that several previous microarray analyses carried out after RF exposure failed to detect any significant changes in the gene expression in normal peripheral [32] or CNS glial cells [33] or cancer cell models.

To add complexity to the picture, Gerner and co-workers [22], comparing the proteomic in Jurkat T-cells and human fibroblast, indicated that a strict relation between metabolic state and responsiveness to RF-EMF exists that implies a higher sensitivity of growing organisms. Moreover, these authors hypothesize that disturbances of hydrogen bonds might play a crucial role to sustain alteration in protein profile after RF active cells to determine its bioeffects.

Cell architectural elements, such as a membrane or a cytoskeleton, can be considered a possible target for EMF radiation damage. In 2004, the REFLEX study, Quality of Life and Management of Living Recourses, funded by the European Union for the evaluation of risk of potential environmental hazards of *in vitro* electromagnetic field exposure, stated that RF-EMF increased microtubular damage and the number of DNA strand breaks in HL-60 cells. Microtubules are macromolecular structures consisting of tubulin heterodimers and are present in almost every eukaryotic cell. It has been demonstrated that 900 MHz interferes with the polymerization process *in vitro* to alter microtubules structure [34]. Recently, however, Speit [35] failed to reproduce genotoxic effects of RF-EMF in HL-60, adding more uncertainty about the real effect of EMF on cell viability.

### 3.2. The Effect of RF on Neuronal Cell Activity

The interest in RF exposure effects on the central nervous system has grown in parallel with mass technology applications. Given the proximity to the area involved in mobile phones handling, several studies analyze cells from facial districts such as eyes and nose, which are doors to the nervous system. In particular, no effect of exposure to a very high SAR (20 W/kg) was observed when retinal ganglion cells were exposed to three mobile phone frequencies (GSM-900, GSM-1800, UMTS). The eye represents an easy target for RF and the damage could compromise not just local tissue but also general visual CNS function. However electrophysiological evidence from single cell recording could not determine any frequency, or intensity or distance-related damage after RF exposure [36]. Similarly, no change was detected in physiological parameters in the nasal area [37] or in the oral mucosa cells both spatially close to cell phone EMF exposure [38]. Regarding neuronal networking, Moretti [39] published a report about the thermal effect of short-term exposure to GSM-1800 on neuronal firing by measuring the spontaneous electrical activity *in vitro*. In this case, the increase of firing activity detected was ascribed to the increase of localized temperature that might influence general EEG and neuronal tissue functions *in vivo*. On the other hand, 1 C increase in temperature after EMF exposure did not elicit activation of heat shock protein in human brain cells [40].

One of the most frequent findings of *in vitro* models after EMF-RF exposure is the increase of oxidative stress-related events that lead to cell damage. This event is causally related to neurodegeneration that underlie severe pathologies such as Alzheimer’s disease or Parkinson’s. Pilla [41] investigated the stress-related effect of RF-EMF pulse exposure on dopaminergic MN9D cultures. The increase of intracellular nitric oxygen (NO) level after exposure was related to the activation of Ca/CaM-dependent constitutive nitric oxide synthase. The CaM/NO/cGMP signaling pathway activates a rapid response cascade that in turn influences EMF on Cam/NO pathways, which can modulate CNS response to inflammation and ischemia and interfere with the ability of the brain to restore post-traumatic functions. Another mechanism to regulate neuronal function is calcium homeostasis, suggested to be a mediator of cell response to RF exposure [42]. While O’Connor [43] found no effect on calcium homeostasis in cultured hippocampal neurons or peripheral human endothelial cell exposed to 900 MHz GSM-modulated RF fields (SARs of 2 W/kg to meet guidelines set by ICNIRP), a growing bulk of literature indicates calcium-related machinery as a potential mediator for EMF effect, both *in vitro* and *in vivo*. Maskey [44,45] hypothesized that repeated five-hour sessions of daily exposure to rats of 835 MHz (SAR = 1.6 W/kg) might alter permeability of cell membrane in hippocampal pyramidal cells, thereby jeopardizing neuronal connectivity.

Regarding the peripheral system, oxidative stress is a critical factor that also interferes with immune system cell viability. Peripheral blood mononuclear cells are a critical component of the immune system that fight infection and adapt to intruders and are also involved in aging and CNS neurodegeneration. EMF-RF induces lipid and protein expression changes leading to reactive oxygen species (ROS) production and caspase-3-dependent apoptosis through weakening mitochondrial membrane potential [46].

What emerges from these reports, and several earlier ones, is that RF exposure is bad for cell survival and that there are some districts such as the brain, in which cells are extremely sensitive to RF exposure. However, we would like to point out a few examples that might suggest otherwise.

### 3.3. To Adapt to Survive

One of the most interesting findings about RF is that exposure to a relatively low intensity of RF-EMF (adaptive dose) can prompt a surviving response in cells when subsequently undergoing potentially lethal insults. This preservative effort is known as adaptive response (AR). It has been described in human lymphocytes [47,48] where an adaptive dose of 1950 MHz RF UMTS (universal mobile telecommunication system) signal was delivered for 20 h with a SAR of 1.25 W/kg. The following treatment with mitomycin C (48 h with 100 ng/mL) significantly reduced genotoxicity with respect to sole mitomycin controls. Zeni *et al*. [49] showed that AR effect was found only in cells that were in S phase at the time of the first adaptive dose exposure, while no effect was found in G0- and G1-phase cells, suggesting a different genotoxicity susceptibility in relation to active cell cycle phases. Similar results were described when lymphocytes pre-exposed to RF exhibited resistance to the genetic damage induced by subsequent exposure to X-rays (XR), an ionizing physical mutagen that induces predominantly strand breaks in DNA [47]. In human promyelocytic leukemia HL-60 cells pre-exposed to 900 MHz RF (0.25 μW/kg average SAR) and subsequently treated with doxorubicin, [50] Jin described a reduction in apoptotic rate with alteration in mitochondrial membrane potential and intracellular calcium and magnesium homeostasis. Although the intracellular mechanism that sustains adaptive response is not fully unveiled the beneficial exposure to RF are worth further investigations given the everyday combined exposures to different EMF sources, as well as the simultaneous exposure to EMF and other factors such as chemicals, noise, stress, *etc*. that could interfere with cell function.

We have reviewed above some of the recent relevant studies on the mechanism involved in DNA damage and repair *in vitro*. Limitation on interpretation of the findings still remains mostly related to experimental conditions and data managing [51]. To date, it appears that the puzzle of intracellular mechanisms involved in EMF-RF exposure cell response is not in any way completed. *In vivo* studies, however, might provide a deeper view of the functional effect of RF exposure to predict the degree of risk for population health.

## 4. The Effect of RF Exposure *in Vivo*: New Insights on Biosystem Functions

Studies carried out *in vivo* on animal models are extremely valuable as the protocols can be designed to apply a rigorous and controlled procedure of exposure for longer time, gaining insights on chronic exposure that better predict the degree of risk for population health. Selected *in vivo* models have been recently used to suit specific scientific questions. For example, *Eisenia Fetida* (Oligochaeta, heartworm) was selected for standardized assays to evaluate ecological risk in terrestrial ecosystems [52]. This model has been validated for toxicological endpoints of genotoxicity such as DNA damage, anti-oxidative enzyme activities, as well as lipid peroxidation. After only 2 h of exposure to 900 MHz EMF at the power density of 23Vm-1, damage to protein, lipids and DNA was detected. Another easily amenable *in vivo* model is *Drosophila melanogaster* used to study DNA fragmentation after 900 MHz exposure through its reproductive ability [40]. To investigate the effect of RF exposure on morphology, biology and functions of mammalian brain and neuronal network functions [53,54] rodents are intensively used. In 2011, Ntzouni *et al*. [55] carried out a study in order to investigate whether short-term memory is affected by ordinary mobile phone exposure. Likewise for many other perturbing events occurring during memory formation, even for RF exposure, timing is crucial to the final outcome. RF exposure shortly after Object Recognition Task (the ability to judge a previously encountered item) deeply affects memory formation, perturbing trace consolidation. In relation to memory formation and storage, one of the most important subcortical areas of CNS is the hippocampus. Pyramidal cells constitute the architectural and functional landmark of *Cornus Ammonis* and dentate gyrus. Excitation in these cells is regulated by intracellular and extracellular calcium homeostasis. RF radiation at 835 MHz (SAR value of 1.6 W/kg) affected calbindin and calretinin, inducing a progressive loss of pyramidal cells in mice exposed to three hours of acute treatment [45]. Moreover, Ammari *et al*. [56] previously indicated that metabolic functions of cytochrome C oxidase were affected by RF 6 W/kg GSM 900 MHz in the brain and in particular in the perirhinal and entorhinal cortex, impinging on memory formation. In this view, it is striking how relatively few studies were carried out on the effect of RF on neurotransmitters [57]. Neurotransmitters such as serotonin (5HT) and dopamine orderly regulate acquisition, memory formation and storage in selected districts of the brain. After up to four months of daily RF exposure (1800 MHz, SAR 0.843 W/kg), adult rats increased serotonin and decreased dopamine levels in the hippocampus to affect memory and learning.

### 4.1. Physiopathology of the Brain

The involvement of RF in several neuropathologies has received wide consideration [58]. Among the neurodegenerative diseases, Alzheimer’s disease (AD) has long been associated with RF exposure. AD is in fact characterized by the presence of well-established morphological (neurofibrillary tangles and β amyloid deposition in cortical and hippocampal neurons) and functional (behavioral and memory alterations) biomarkers. One of the most relevant features of Alzheimer’s seems to be the increased reactive oxygen species (ROS) production and related inflammation of cortical and subcortical tissue in AD brains. There are many reports of ROS increase after RF exposure in the brain [59,60] that in turn give rise to a cascade of molecular events involving anti-oxidative enzyme activities, protein kinase C, creatine kinase and finally the pro-apoptotic enzyme caspase 3. RF-dependent expression of reactive astroglia was also detected [56]. There seems to be a causal relation between AD development and RF exposure; however, few reports suggested a beneficial influence of RF on this pathology. Although recent evidence would suggest otherwise, [61,62] it has been demonstrated that chronic pulse application of RF can revert both deposition of β amyloid and ameliorate cognitive deficit in mice [63]. A similar effect was also obtained in either wild type and mutated mice [64], where RF exposure increased mitochondrial activity and reduced cerebral blood fluid and seemed to facilitate soluble β amyloid removal from the CNS district. Interestingly, Banaceur and co-workers [65] pointed their attention to another aspect not yet fully investigated namely Wi-Fi radiofrequencies (2.40 GHz SAR 1.6 W/kg). 3× Tg-AD transgenic mice [66] were exposed for 28 consecutive days and tested for cognitive and behavioral tasks. It appears that Wi-Fi exposure might determine a positive cognitive interference and a beneficial influence on anxiety. The authors are set to explore the molecular pathways that underlie these findings.

Few reports concern Parkinson’s disease (PD) and RF exposure. Parkinson’s is another severe neurodegenerative disease and is caused by a degeneration of dopaminergic neurons in the *Substantia Nigra* of the midbrain. A possible causal link between RF and PD has been suggested [67,68]. Dopamine expression and the expression of those enzymes that participated in its metabolism, such as monoaninoxidases (MAOs), have also been investigated after RF exposure in rats [69]. Moreover, abnormal aggregation of α-synuclein plays a crucial role in Parkinson’s disease pathogenesis and its toxicity. The physiological function of α-synuclein remains uncertain; however, α-synuclein protein deposition in Lewy bodies is considered as a biomarker of PD because its accumulation, even to a small extent, may be a risk factor for neurodegeneration. In cultured neuron-enriched mixed cortical cell cultures from the brains of rat embryos, the expression of α-synuclein was found to be down-regulated by 900 MHz acute exposure [70]. Long-term whole body irradiation of Balb/c mice showed altered protein expression in cerebellum, hippocampus, and frontal lobe. Most relevantly, α-synuclein expression, together with glial fibrillary acidic protein (GFAP), glia maturation factor beta (GMF) and apolipoprotein E (apoE) expression, was altered, indicating impaired neuronal plasticity. Moreover, heat shock proteins, and cytoskeletal proteins also varied significantly, suggesting possible structural and morphological alterations in the RF exposed brain [71]. The real effect of RF exposure on PD induction and development remains uncertain.

In relation to the CNS, it is worth mentioning a few studies on the effect of RF on neuronal blood barrier permeability. Blood-brain barrier (BBB) and blood-retinal barrier [72] are safety mechanisms that regulate molecular exchanges between blood circulation and neuronal tissues. Variation of barrier permeability may occur as a consequence of pathologies or traumas, such as after stroke. However, an increase of permeability is seldom required for drug delivery and substance exchanges. BBB permeability can be transiently increased by pulsing low frequency electromagnetic field [73]. *In vivo*, GMS 900 MHz whole-body exposure of rats for 2 h per day for 7 consecutive days (SAR up to 14 mW/kg) increases BBB permeability that lasts up to 14 days after treatment. More importantly in these settings, no neuronal loss was described [74]. Although transient opening of the BBB could be exploited for therapeutic purpose, the benefit/risk balance is to be cautiously evaluated.

Concern has been raised about the potential negative influence of RF on a developing brain [75]. Brain development in mammals is time and growth factor-dependent and extremely sensitive to external insult that might compromise future network functions. It has been shown that pre- and post-natal exposure of rat embryos to commercial mobile phone RF induced an increase of neurodegeneration in Purkinje cells in the cerebellum of rats assessed during adulthood [76]. Moreover, supplementation of a lycopene (natural antioxidant)-rich diet partially prevents neurodegeneration coincidental with caspase 3-dependent apoptotic cascade. Although no specific indication of the exposure time was given, other than “pre and post natal period”, these results are quite impressive and at the same time alarming if scaled up to the human conditions.

Equally noteworthy is the effect of RF 900 MHz long-term application on juvenile rats [77] that appeared to be more susceptible to oxidative stress metabolism measured in peripheral investigated tissues and blood. This effect is likely due to the altered level of blood glutathione (GTH). Similarly, *in vitro* culture of bone marrow cells from mature rats showed that cytogenotoxic damage was more remarkable than in adults [78]. Melatonin is another important molecular player regulating brain physiology. Several groups investigated the disruptive effect of EMF on the production of the hormone melatonin by the CNS pineal gland, which controls the body’s circadian rhythms [79]. Melatonin production is sensitive to light and to exposure of a different range of EMF (recently reviewed in [80]). This evidence indicated melatonin as a possible target for sleep-related disorders. However, there is a lack of solid evidence to suggest a significant impact of EMF exposure on sleep quality in animal models or in the population [81]. Melatonin suppression by EMF has been indicated as a potential risk factor for breast cancer, though no direct tumorigenic effect was observed. This hormone activates specific receptors, namely MT1 [82]. However, it also acts as a natural free radical antioxidant scavenger. This would explain its beneficial effect in reducing EMF-induced damage in breast cancer model [83] and its neuroprotective rescue after RF oxidative stress induction in neuron primary culture [84]. Interestingly, melatonin has been reported to play a relevant role in regulation of human newborn protection in enhanced environmental oxidative stress (OS) present at time of birth. Preterm babies are highly prone to OS and to the toxic effect of free radicals, as for example those involved in perinatal brain lesion, as babies’ defense homeostatic neuroprotective mechanisms are not fully developed. Therefore, the impact of EMF exposure of newborn babies to EMF derived from nursing incubators cannot be overlooked [85]. A slight transitory increase in urine metabolite indicates an overproduction of melatonin in babies removed from the incubators with respect to control babies; however, the real implication of this finding is not yet fully understood.

### 4.2. Can We Trust Wi-Fi(delity)?

Recently, Wi-Fi exposure (1 h per day, for 36 days) effect was measured in reproductive organs of adults and embryonic development of offspring. In this case, although thoroughly investigated, both morphologically and molecularly, no adverse effect was noted in either the pups or the reproductive functions of parents [86,87]. Laudisi *et al.* [88] found that whole-body exposure to Wi-Fi (2450 MHz 4 W/kg) during the entire pregnancy of C57BL/6 mice did not interfere with the number of newborn or lymphocyte B maturation in the offspring thymus. Moreover, T-cells isolated from pups showed no difference in proliferation with respect to the placebo animals. The complexity of the picture does not allow for the underestimation of possibly undiscovered adverse effects on developing biological systems [89], and these data must be evaluated in light of involuntary EMF environmental pollution exposure to children.

## 5. The Effects of RF Exposure on Humans

The International Agency for Research on Cancer (IARC) at the WHO evaluated the carcinogenic risk for humans from prolonged exposure to RF, naming the risk as “possible” (2B) [90,91]. However, recent evidence of long-term exposure studies on tumors might suggest endorsing a stricter set of criteria to raise the risk up to group 1 according to the IARC classification. The USA Federal Communications Commission’s (FCC’s) regulations for conducting environmental reviews under the National Environmental Policy Act (NEPA) recently issued a final document for regulating safety, economical and social aspects of the RF usage and the compliance with its limits, including SAR.

To date, RF exposure remains a crucial issue among the general public as numerous subjects are reporting that they suffer from hypersensitivity to exposure [92,93]. Scientists are trying to answer all calls for specific categories’ interaction with environmental or professional RF exposure. Geographical location [90,94,95], occupational groups [96,97], sections of the population [98] such as children and youngsters [99,100] or pregnant women [101] are only several elements that have been investigated in a cohort of voluntary subjects participating in research or clinical studies, or considered in informative epidemiologic meta-analysis [102] surveys. Moreover, the impacts of diagnostic devices such as magnetic resonance scanners that emit RF were analyzed [103]. Given the delicate matter, it is not infrequent to find comments and rebuttals between different research groups. The vast diversity of protocols and the different quest of researchers together with the ever-increasing alert on these issues make the systematic review of all of the new findings virtually unfeasible. However, among recent studies, those on voluntary cohorts are perhaps the most interesting. We selected a few relevant examples on the investigation of how RF might impinge on everyday life of selected segments of the populations. In particular, a study that investigated mobile phone use in youngsters is quite alarming. Alsanosi [104] found that one hour of continuous use of the mobile phone immediately caused hearing dysfunction.

General physiological state is also under investigation. For example, Parazzini *et al.* [105] show that repeated exposure to GMS cell phone 900 MHz does not interfere with non-linear dynamics of heart-rate variations in healthy volunteers.

Regarding brain stimulation, many experiments were carried out to establish interference of RF and EEG in relation to cognitive functions [106] as well as with sleep [107,108]. Recently, a group of Japanese youngsters underwent a test to evaluate the effect on EEG of mobile phone emission for three hours prior to sleep. Both subjective (headache, dizziness) or objective evaluation parameters (EEG spectra) did not differ in the placebo-exposed group [109].

### 5.1. Diagnostic Benefits and Occupational RISK

Among the numerous sections of the population that occupationally encounter RF exposure, workers using MRI equipment hold a special place. MRI is an imaging technique that employs strong static, gradient, and radiofrequency magnetic fields. It can image soft tissues—unobstructed by bone—with enhanced contrast. In a clinical MRI system operating at 1.5 T, because of its design, it is unlikely that radiological staff would be exposed to significant RF fields. The gradient field is pulsed rapidly in time and is a function of the imaging technique and design of the MRI system. Recently, the demand for increased spatial resolution and high signal-to-noise ratio from MRI instruments has prompted the development of systems using much higher static magnetic fields (greater than 11 T). This development has led to the use of higher RF frequencies for MRI, which, in principle, not only can augment the amount of RF power deposition inside the patient’s body, but also increases the EMF exposure for workers using MRI in the hospital environment and workers employed for supporting, servicing, developing and manufacturing this equipment [5]. We report here a few examples of surveys to evaluate and confine these risks. In Schaap *et al*. [110], a thorough investigation identified the structure and the professional categories most likely to be involved in the use of MRI that include people using MRI for clinical diagnostics, MR-guided medical interventions on patients. Bongers *et al.* [111] carried out a study by interviewing and evaluating self-assessing workers employed in production and development of MRI systems. To evaluate the level of RF exposure and generate a predictive algorithm universally applicable to model and predict the level of exposure and the effect people working with and around MRI systems might experience. This kind of information constitutes the basis for improvement in design, application and risk management associated with the use of MRI.

### 5.2. Modelling

The exposure reference levels are derived using simplified anatomical models to conservatively relate the occurred field levels with the basic restrictions. Although it is assumed that the basic restrictions are satisfied if the electromagnetic field exposure is below the reference levels, further measures need to be taken to indicate the compliance with safety limits if the occurred fields violate the reference levels. These measures can be, for instance, direct measurements of the SAR or simulations of the SAR or of the induced currents using anatomical high-resolution models of the human body. Indeed, to better describe the risk of exposure, increasingly sophisticated algorithms and mathematical simulation must take place [112–115]. Recently, SAR for RF was modelled on a 3D anatomical eye model. Eyes seem to be the most threatened by such exposure and most sensitive to temperature increase that often cause or worsen cataracts. Similarly, head phantoms are used to estimate the 3-D SAR distribution of four realistic models of mobile phones [116]. This kind of information might be exploited for implementing safer mobile phone design [117]. Whole-body phantom was used [118] to experimentally assess the whole-body average SAR in a complex indoor environment. All of these predictive methods are validated through numerical simulations and may provide tools to determine compliance with the safety guidelines for SAR levels.

## 6. Conclusions

It is virtually impossible to account for all of the potential situations in which we encounter casual or expected EMF exposure in our daily life. Moreover, although regulated by physical and mathematic laws, it is quite tricky to describe the variable interactions between RF and biological systems.

One can envisage that EMF “speaks” to each organism and each cell with a different language. The answer to that call can potentially induce protein modification, ion exchanges and nucleic acid conformational changes that might cause positive, adaptive or destructive effects and the modulation of EMF can determine the benefit or the severity of the outcomes. Numerous works indicated that tuning the use of pulsating low and extremely high frequency (EMF) trains of stimulation might be used in cancer treatment [1] as well as in regenerative medicine [119,120] where cells can be induced to differentiate with minimal manipulation and without pharmacological treatment or gene modification [121]. One of the most exciting findings is that using the ion cyclotron resonance of different elements, *i.e.*, calcium (7 Hz 9.2 micro Tesla), it is possible to induce neuronal differentiation, reducing the carcinogenic phenotype [122]. We foresee the possibility that this tuning can be achieved also with RF in the range of 900 MHz, as some positive reposts on memory enhancement would suggest.

It is therefore important that at least in research, the standardization of protocols, reproducibility of results and unbiased interpretation take place. We attempted an overview of the most recent advances in understanding the nature and the possible functional impact of the effects induced by RF exposure on living beings. We discussed the molecular path finding of the *in vitro* investigation as well as the functional complexity of *in vivo* experimental designs. The overwhelming amount of the sometimes contradictory incoming data about potential damage in humans has been briefly considered here as regulatory agency guidelines continuously monitor and regulate the issue.
